# An atlas on risk factors for multiple sclerosis: a Mendelian randomization study

**DOI:** 10.1007/s00415-020-10119-8

**Published:** 2020-07-29

**Authors:** Shuai Yuan, Ying Xiong, Susanna C. Larsson

**Affiliations:** 1 Unit of Cardiovascular and Nutritional Epidemiology, Institute of Environmental Medicine, Karolinska Institutet, Stockholm, Sweden; 2 Department of Public Health Sciences, Karolinska Institutet, Stockholm, Sweden; 3 Department of Surgical Sciences, Uppsala University, Dag Hammarskjölds Väg 14B, Uppsala, Sweden

**Keywords:** Multiple sclerosis, Risk factors, Prevention strategy, Mendelian randomization

## Abstract

**Objectives:**

We conducted a systematic review and wide-angled Mendelian randomization (MR) study to examine the association between possible risk factors and multiple sclerosis (MS).

**Methods:**

We used MR analysis to assess the associations between 65 possible risk factors and MS using data from a genome-wide association study including 14 498 cases and 24 091 controls of European ancestry. For 18 exposures not suitable for MR analysis, we conducted a systematic review to obtain the latest meta-analyses evidence on their associations with MS.

**Results:**

Childhood and adulthood body mass index were positively associated with MS, whereas physical activity and serum 25-hydroxyvitamin D were inversely associated with MS. There was evidence of possible associations of type 2 diabetes, waist circumference, body fat percentage, age of puberty and high-density lipoprotein cholesterol. Data of systematic review showed that exposure to organic solvents, Epstein Barr virus and cytomegalovirus virus infection, and diphtheria and tetanus vaccination were associated with MS risk.

**Conclusions:**

This study identified several modifiable risk factors for primary prevention of MS that should inform public health policy.

**Electronic supplementary material:**

The online version of this article (10.1007/s00415-020-10119-8) contains supplementary material, which is available to authorized users.

## Introduction

Multiple sclerosis (MS) is an inflammatory demyelinating disease of the central nervous system and a leading non-traumatic cause of disability among young adults of northern European ancestry [1]. Even though epidemiological studies have uncovered several modifiable risk factors for MS, such as serum vitamin D levels [2] and body mass index [3], the overall etiological basis of MS is poorly understood [4]. A recent umbrella review found consistent evidence supporting the associations of Epstein-Barr virus infection and smoking with MS risk [1]. However, the role of other environmental factors and internal conditions for MS risk have been scarcely investigated. In addition, it is unclear whether the associations reported by traditional observational studies are causal due to potential confounding, reverse causality and misclassification of such studies.

Mendelian randomization (MR) is an analytical approach that utilizes genetic variants, generally single nucleotide polymorphisms (SNPs), as instrumental variables for an exposure to diminish confounding and reserve causality, thereby strengthening the causal inference of an exposure-outcome association [5]. The rationale of minimizing confounding in MR studies is that genetic variants are randomly allocated at meiosis, and therefore, one trait is generally unrelated to other traits. Reverse causality can be avoided since genetic variants are fixed and, therefore, cannot be modified by disease onset and progression [5]. There are three key assumptions for MR analysis [5]. First, the genetic variants proposed as instrumental variables should be associated with the risk factor of interest. Second, the used genetic variants should not be associated with potential confounders. Third, the selected genetic variants should affect the risk of the outcome (e.g. MS) merely through the risk factor. Exploiting different summary genetic sources for an exposure and outcome, the two-sample MR approach infer the exposure-outcome causality with improve statistical power and less confounding bias [6].

The aim of the present study was to systematically appraise the evidence of causal associations between possible risk factors and MS using the two-sample MR design. For exposures that cannot be instrumented by genetic variants, we additionally obtained data from the latest meta-analyses through a systematic review of the literature.

## Methods

### Study design overview and potential risk factor identification

**Fig. 1:**
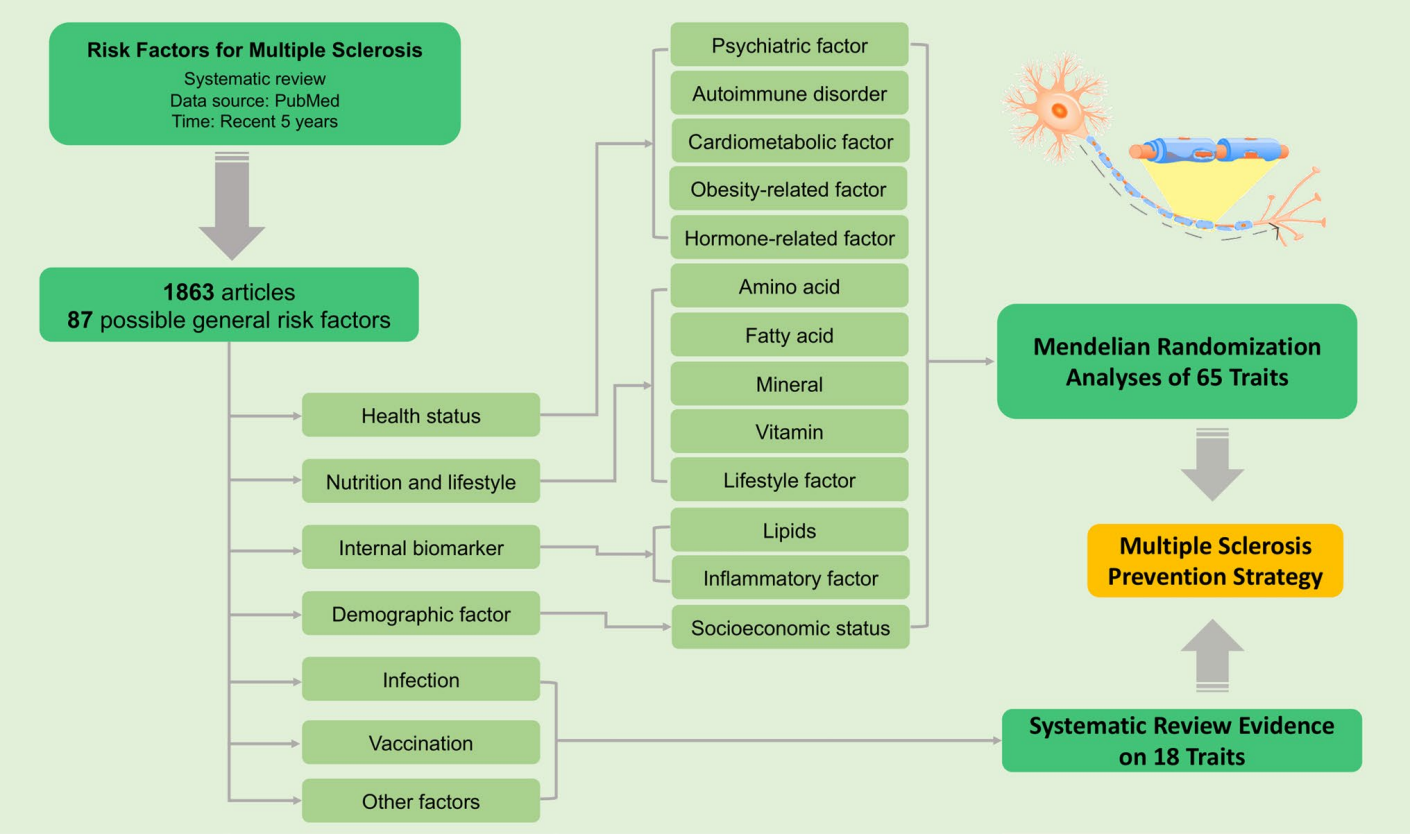
Overview of study design

The overview of the study design is displayed in Fig. 1. First, we conducted a systematic review in the PubMed database to identify possible risk factors for MS. In total, 1863 studies published in recent 5 years were screened, and 87 general risk factors were pinpointed (Supplementary Table 1). After excluding traits without suitable genetic instruments or limited genetic instruments (SNPs < 3), a total of 65 possible risk factors were included in the MR analyses. In addition, we included 18 risk factors in the systematic review.

### Mendelian randomization study

#### Instrumental variable selection

Instrumental variables for the 65 exposures were identified from genome-wide association studies (GWASs). SNPs at the genome-wide significance threshold (*p* < 5 × 10^–8^) were proposed as instrumental variables. To mitigate against co-linearity between included SNPs, we excluded SNPs in linkage disequilibrium (*R*^2^ ≥ 0.01) and retained SNPs with the strongest effect on the associated trait. SNPs in the *MHC* gene region, which is strongly associated with MS, were removed from analyses to exclude possible pleiotropy. The variance explained by used SNPs for individual risk factor was either extracted from the original GWAS or estimated based on minor allele frequency, beta coefficient for minor allele and standard deviation (SD) for the risk factor. Information of the data sources as well as the number of SNPs used, and the variance explained by the SNPs is presented in Table 1. Other information, such as instrumental variable selection and unit for each trait, is available in Supplementary Table 2.

**Table 1:** Data sources and instrumental variables used for exposures included in the MR analyses

Exposure	Cases or sample size	Controls	Population	SNPs	Used SNPs^c^	Variance (%)^d^	*F* statistic	PubMed ID
Psychiatric factor								
Lifetime anxiety disorder	25 453	58 113	European	5	5	0.5^e^	84	BioRxiv
Major depressive disorder	414 055	892 299	European	95	91	1.8^d^	252	30718901
Sleep duration	446 118	NA	European	76	3	0.7^e^	41	30846698
Short sleep (< 7 h)	106 192	305 742	European	26	1	1.2^d^	192	30846698
Long sleep (> 9 h)	34 184	305 742	European	6	6	0.7^d^	399	30846698
Insomnia	397 972	933 038	European	238	206	2.6^e^	149	30804565
Morningness	372 765	278 530	European	329	313	11.9^d^	267	30696823
Restless leg syndrome	15 126	95 725	European	20	20	11.7^e^	734	29029846
Autoimmune disorder								
Type 1 diabetes	9934	16 956	European	18	17	6.7^e^	107	21980299
Latent autoimmune diabetes in adults	2634	5947	European	3	3	0.7^d^	20	30254083
Allergic rhinitis	59 762	152 358	European	33	32	4.7^d^	317	30013184
Asthma	10 549	47 146	European	18	16	13.7^d^	509	30552067
Eczema (atopic dermatitis)	18 900	84 166	European	20	19	7.2^d^	400	26482879
Rheumatoid arthritis	18 136	49 724	European	27	24	8.1^d^	221	24390342
Cardiometabolic factor								
Type 2 diabetes	74 124	824 006	European	179	160	1.0^d^	51	30297969
Fasting glucose^a^	133 010	NA	European	35	35	4.8^d^	192	22885924
Fasting insulin^a^	133 010	NA	European	18	18	1.0^d^	75	22885924
Hemoglobin A1c^a^	up to 159 940	NA	Mixed	48	45	4.7^e^	164	28898252
Hemoglobin^a^	135 367	NA	Mixed	25	17	NA	NA	23222517
Diastolic blood pressure	> 1 million	NA	Mixed	271	262	5.3^e^	21	30224653
Systolic blood pressure	> 1 million	NA	Mixed	229	222	5.7^e^	26	30224653
Coronary artery disease	60 801	123 504	Mixed	45	43	1.7^e^	71	26343387
Peripheral artery disease	36 424	601 044	European	18	17	3.5^d^	1284	31285632
Obesity-related factor								
Birth weight	up to 500 000	NA	European	92	92	2.5^d^	139	30305743
Childhood body mass index	35 668	NA	Mixed	15	15	2.2^d^	53	26604143
Adulthood body mass index	250 000	NA	European	963	963	7.8^d^	22	30124842
Waist circumference	500 000	NA	European	314	314	4.6^d^	77	30305743
Lean body mass	47 227	NA	European	7	7	4.6^d^	325	30721968
Body fat percentage	up to 500 000	NA	European	364	364	5.3^d^	77	30305743
Circulating adiponectin^a^	45 891	NA	Mixed	10	10	NA	NA	22479202
Hormone-related factor								
Age of puberty	up to 370 000	NA	Mixed	339	315	7.4^e^	87	28436984
Age of natural menopause	up to 70 000	NA	European	42	42	NA	NA	26414677
Other factors								
Migraine	59 674	316 078	European	33	33	4.4^d^	524	27322543
Migraine without aura	2326	4580	European	6		7.8^d^	97	22683712
Estimated bone mineral density	426 824	NA	European	993	786	20.7^e^	112	30598549
Uric acid^a^	288 649	NA	European	123	94	5.3^e^	131	31578528
Amino acid								
Carnitine^a^	7824	NA	European	18	18	13.8^d^	69	24816252
Homocysteine^a^	44 147	NA	European	16	13	3.3^d^	94	23824729
Isoleucine^a^	16 596	NA	European	4	4	1.1^d^	46	27898682
Plasma fatty acid								
Docosapentaenoic acid	8866	NA	European	3	3	3.2^d^	98	21829377
Linoleic acid	8631	NA	European	3	3	2.1^d^	62	24823311
Palmitoleic acid	8961	NA	European	5	5	3.5^d^	65	23362303
Stearic acid	8964	NA	European	3	3	2.1^d^	64	23362303
Mineral								
Calcium^a^	39 400	NA	European	7	7	0.9^d^	51	24068962
Magnesium^a^	15 366	NA	European	6	6	1.6^d^	42	20700443
Sodium^b^	446 237	NA	European	47	45	NA	NA	31409800
Potassium^b^	446 238	NA	European	12	12	NA	NA	31409800
Iron^a^	48 972	NA	European	5	5	3.4^d^	345	25352340
Vitamin								
Folate (vitamin B9) ^a^	37 341	NA	Mixed	3	3	0.8^d^	100	23754956
Vitamin B12^a^	45 576	NA	Mixed	10	9	4.5^d^	215	23754956
Vitamin D^a^	121 640	NA	European	7	7	5.3^d^	972	29343764
Vitamin E^a^	5006	NA	European	3	3	1.7^e^	29	21729881
Lifestyle factor								
Alcohol drinking	941 280	NA	European	87	81	2.5^e^	277	30643251
Coffee consumption	375 833	NA	European	15	12	0.5^e^	126	31046077
Smoking initiation	1 232 091	NA	European	349	307	1.0^e^	36	30643251
Physical activity	377 234	NA	European	6	6	0.8^d^	507	29899525
Serum lipids								
High-density lipoprotein cholesterol^a^	188 577	NA	Mixed	68	68	1.6^d^	45	24097068
Low-density lipoprotein cholesterol^a^	188 577	NA	Mixed	58	58	2.4^d^	80	24097068
Total cholesterol^a^	188 577	NA	Mixed	74	74	2.6^d^	68	24097068
Triglycerides^a^	188 577	NA	Mixed	37	37	2.1^d^	109	24097068
Inflammatory biomarker								
Tumor necrosis factor^a^	30 912	NA	European	3	3	0.6^e^	62	–
C-reactive protein^a^	204 402	NA	European	55	55	7.0^e^	280	30388399
Immunoglobulin E^a^	6819	NA	European	3	3	1.6^e^	37	22075330
Socioeconomic status								
Educational level	1 131 881	NA	European	1197	789	12.0^d^	129	30038396
Intelligence	269 867	NA	European	230	201	5.2^d^	64	29942086

Variables without controls information are continuous variables

*NA* not available; *SNP* single nucleotide polymorphism

^a^Measurement of these indicators was based on serum levels

^b^Measurement of these indicators was based on urinary levels

^c^Numbers of SNPs used in the present Mendelian randomization analyses

^d^Variance estimation was based on the formula *R*^2^ = 2 × MAF × (1 − MAF) × (beta/SD)^2^ (MAF indicates minor allele frequency; beta estimation was based on MAF; and SD was one for continuous traits with SD unit or binary traits without variance information in original genome-wide association studies

^e^For continuous traits that were not scaled into SD unit, variance explained was extracted from the original genome-wide association studies. For binary traits with variance information in original genome-wide association studies, variance was extracted from the paper directly

### Multiple sclerosis genotyping data

Summary-level statistics for the associations of 65 risk factor-associated SNPs with MS were extracted from the discovery stage of a GWAS with 14 498 MS cases and 24 091 controls of European ancestry from 11 countries [7]. Beta and standard error for identified SNPs had been obtained by logistic regression analysis with adjustment for five population principal components. MS cases were diagnosis by Neurologists familiar with multiple sclerosis in accordance with recognised diagnostic criteria that employ a combination of clinical and laboratory-based para-clinical information. Detailed cases ascertainment criteria in every included area were specified in the published GWAS of MS [7]. Overall and country-specific disease-related features, such as sex ratio, onset age, age at examination and disease severity, is displayed in Supplementary Table 3.

### Statistical analysis

The association between individual risk factor and MS risk attributable to each SNP was estimated with the Wald method. The ratio estimates for every used SNPs for one trait were combined by using the multiplicative random-effects inverse-variance weighted (IVW) meta-analysis method [8]. We used the weighted median approach as sensitivity analysis, which can provide a consistent estimate with the prerequisite that more than 50% of the weight in the analysis comes from valid instrumental variables [8]. The MR-PRESSO approach was used to correct for possible pleiotropic effects. The MR-PRESSO test detects possible outliers and provides estimates after removal of outliers, thereby correcting for horizontal pleiotropy [9]. Heterogeneity across used SNPs for a trait was measured by the Cochranes’s Q statistic and possible pleiotropy was detected by MR-Egger regression model with *p* for intercept ≤ 0.05 [8]. To assess the strength of the instrumental variables, F-statistics was estimated based on sample size, numbers of SNPs used, and variance explained by included SNPs [10]. Power estimation was based on a web-tool [11] and is shown in Supplementary Table 2. Odds ratios (ORs) and 95% confidence intervals (CIs) of MS were scaled to one-unit increase in corresponding units for different traits. All statistical analyses were two-sided and performed using the mrrobust package in Stata/SE 15.0 and TwoSampleMR in R 3.6.0 software. Associations with *p* value < 0.05 in both IVW-random effects and MR-PRESSO models were deemed as robust associations and associations with *p* < 0.05 in either IVW-random effects or MR-PRESSO model and in the same direction across all analyses were regarded as suggestive associations.

### Systematic review

With regard to risk factors not suitable for MR analysis, we conducted systematic reviews to obtain the latest meta-analysis including the most studies. Systematic reviews were carried out on 18 risk factors. We extracted published information, number of included studies, sample size and risk estimates. Detailed information and search strategies are documented in Supplementary Table 4.

## Results

### Mendelian randomization

Among 65 possible risk factors, four traits, including childhood and adulthood body mass index, serum 25-hydroxyvitamin D and physical activity, were robustly associated with risk of MS. There were suggestive associations with 5 risk factors, including type 2 diabetes, waist circumference, body fat percentage, age of puberty and high-density lipoprotein cholesterol.

### Health status

Six out of 36 health status-related risk factors were associated with MS (Table 2). Specifically, liability to type 2 diabetes, childhood and adulthood body mass index, waist circumference and body fat percentage were positively associated with MS risk, whereas age of puberty was inversely associated with risk. Even though there was heterogeneity in the above analyses, no indication of pleiotropy was revealed in MR-Egger regression analysis (all *p* > 0.05). The other 30 factors showed limited evidence for an association with MS risk.

**Table 2:** Associations between exposures and multiple sclerosis in Mendelian randomization analyses

	IVW-random effects method			Weighted median method			MR-PRESSO			Cochrane’s Q	*P*_het_	*P*_intercept_
Exposure	OR	95% CI	*p*	OR	95% CI	*p*	OR	95% CI	*p*			
Psychiatric factor												
Lifetime anxiety disorder	0.95	0.76–1.18	0.617	1.03	0.86–1.25	0.720	1.02	0.85–1.22	0.840	13.0	0.011	0.673
Major depressive disorder	1.03	0.79–1.35	0.812	0.99	0.75–1.31	0.948	1.04	0.83–1.29	0.753	220.2	< 0.001	0.723
Sleep duration in hour	1.00	0.99–1.01	0.970	1.00	0.99–1.01	0.523	1.00	0.99–1.01	0.846	138.4	< 0.001	0.378
Short sleep (< 7 h)	1.31	0.99–1.74	0.055	1.06	0.76–1.48	0.741	1.31	0.99–1.74	0.068	39.7	0.023	0.621
Long sleep (> 9 h)	1.09	0.72–1.65	0.677	1.15	0.81–1.63	0.429	1.03	0.78–1.36	0.848	12.7	0.026	0.237
Insomnia	1.00	0.88–1.13	0.950	1.06	0.97–1.15	0.235	1.07	1.00–1.15	0.050	1014.8	< 0.001	0.809
Morningness	0.98	0.85–1.13	0.780	0.92	0.82–1.02	0.111	0.95	0.87–1.03	0.189	1282.4	< 0.001	0.134
Restless leg syndrome	0.98	0.93–1.04	0.535	1.00	0.92–1.08	0.985	0.98	0.93–1.04	0.542	20.2	0.384	0.561
Autoimmune disorder												
Type 1 diabetes	1.09	0.93–1.29	0.288	1.00	0.91–1.09	0.963	1.05	0.99–1.13	0.162	192.2	< 0.001	0.318
Latent autoimmune diabetes in adults	0.99	0.59–1.64	0.962	1.07	0.97–1.17	0.173	–	–	–	89.5	< 0.001	0.268
Allergic rhinitis	0.85	0.59–1.22	0.365	1.00	0.82–1.21	0.972	0.88	0.77–1.02	0.100	324.5	< 0.001	0.361
Asthma	0.96	0.82–1.12	0.598	0.97	0.87–1.08	0.554	0.94	0.86–1.03	0.190	88.8	< 0.001	0.089
Eczema (atopic dermatitis)	1.33	0.81–2.18	0.256	1.06	0.88–1.28	0.532	1.31	1.09–1.57	0.018	489.4	< 0.001	0.497
Rheumatoid arthritis	1.05	0.87–1.27	0.598	0.95	0.82–1.09	0.437	1.02	0.90–1.15	0.801	147.2	< 0.001	0.050
Cardiometabolic factor												
Type 2 diabetes	1.08	0.99–1.19	0.089	1.11	1.01–1.21	0.029	1.10	1.04–1.16	5.7 × 10^–4^	737.9	< 0.001	0.978
Fasting glucose	1.14	0.90–1.46	0.282	1.01	0.80–1.27	0.956	1.14	0.85–1.53	0.405	93.0	< 0.001	0.72
Fasting insulin	1.08	0.72–1.61	0.721	1.12	0.72–1.72	0.620	0.90	0.26–3.09	0.743	31.4	0.018	0.268
Hemoglobin A1c	0.86	0.51–1.47	0.590	0.92	0.56–1.53	0.761	1.15	0.76–1.74	0.518	109.7	< 0.001	0.973
Hemoglobin	1.24	0.83–1.84	0.294	1.41	1.02–1.94	0.038	1.41	1.02–1.93	0.061	75.3	< 0.001	0.749
Diastolic blood pressure	1.00	0.98–1.02	0.978	1.00	0.97–1.02	0.906	1.00	0.98–1.02	0.698	476.6	< 0.001	0.519
Systolic blood pressure	1.00	0.98–1.01	0.681	0.99	0.98–1.01	0.386	1.00	0.98–1.01	0.445	340.1	< 0.001	0.475
Coronary artery disease	0.99	0.90–1.09	0.917	1.00	0.92–1.09	0.976	0.97	0.90–1.04	0.358	140.1	< 0.001	0.822
Peripheral artery disease	1.17	0.92–1.49	0.214	1.16	0.93–1.43	0.183	1.08	0.91–1.29	0.387	51.1	< 0.001	0.745
Obesity-related factor												
Birthweight	0.94	0.68–1.30	0.700	0.97	0.76–1.23	0.787	0.97	0.79–1.20	0.773	433.0	< 0.001	0.718
Childhood body mass index	1.23	1.05–1.43	0.010	1.20	0.97–1.48	0.089	1.22	1.07–1.40	0.011	9.4	0.743	0.862
Adulthood body mass index	1.27	1.1.5–1.41	2 × 10^–6^	1.25	1.08–1.44	0.003	1.28	1.16–1.40	2.9 × 10^–7^	1500.9	< 0.001	0.452
Waist circumference	1.20	0.97–1.48	0.096	1.27	1.06–1.53	0.010	1.30	1.14–1.47	7.0 × 10^–5^	1239.9	< 0.001	0.753
Lean body mass	1.06	0.95–1.18	0.311	1.02	0.90–1.15	0.756	1.05	0.94–1.17	0.464	14.7	0.023	0.843
Body fat percentage	1.18	0.97–1.43	0.089	1.27	1.08–1.50	0.004	1.26	1.12–1.43	2.1 × 10^–4^	1345.1	< 0.001	0.909
Circulating adiponectin	1.00	0.83–1.20	0.982	1.03	0.81–1.31	0.807	1.00	0.83–1.20	0.982	9.8	0.369	0.970
Hormone-related factor												
Age of puberty	0.90	0.80–1.01	0.073	0.97	0.88–1.08	0.613	0.90	0.85–0.97	0.004	1224.3	< 0.001	0.128
Age of natural menopause	0.98	0.93–1.03	0.395	1.00	0.96–1.05	0.943	0.99	0.96–1.03	0.771	126.7	< 0.001	0.464
Other factors												
Migraine	1.09	0.93–1.28	0.267	1.10	0.92–1.31	0.283	1.08	0.93–1.24	0.317	64.2	< 0.001	0.838
Migraine without aura	1.03	0.93–1.14	0.574	1.02	0.91–1.14	0.789						0.180
Estimated bone mineral density	1.02	0.95–1.10	0.569	1.11	0.98–1.25	0.089	1.05	0.98–1.12	0.164	1340.7	< 0.001	0.633
Uric acid	1.03	0.92–1.14	0.633	1.00	0.92–1.09	0.952	1.01	0.93–1.10	0.743	265.7	< 0.001	0.621
Amino acid												
Carnitine	0.99	0.90–1.09	0.898	1.07	0.98–1.16	0.120	1.03	0.98–1.08	0.313	42.2	< 0.001	0.206
Homocysteine	0.87	0.68–1.12	0.289	0.87	0.69–1.09	0.227	0.93	0.80–1.08	0.374	24.7	0.010	0.614
Isoleucine	0.98	0.69–1.38	0.892	1.03	0.76–1.39	0.844	0.98	0.69–1.38	0.892	7.8	0.050	0.579
Plasma fatty acid												
Docosapentaenoic acid	1.03	0.71–1.50	0.867	1.05	0.72–1.53	0.805	–	–	–	1.2	0.544	0.476
Linoleic acid	0.99	0.98–1.02	0.762	0.99	0.97–1.02	0.552	–	–	–	2.9	0.235	0.527
Palmitoleic acid	0.96	0.82–1.11	0.571	0.92	0.77–1.10	0.352	0.96	0.82–1.11	0.571	4.9	0.296	0.472
Stearic acid	1.09	0.96–1.21	0.194	1.06	0.93–1.20	0.418	–	–	–	1.3	0.522	0.584
Mineral												
Calcium	0.78	0.33–1.83	0.564	0.88	0.50–1.54	0.644	0.98	0.54–1.77	0.940	18.9	0.004	0.741
Magnesium	1.16	0.94–1.44	0.172	1.19	0.91–1.54	0.199	1.16	0.94–1.44	0.172	1.7	0.789	0.412
Sodium	2.16	1.00–4.68	0.050	1.41	0.56–3.55	0.461	2.16	1.00–4.68	0.050	78.9	< 0.001	0.642
Potassium	0.74	0.13–4.23	0.736	1.39	0.24–8.04	0.713	1.16	0.30–4.50	0.836	22.5	0.021	0.113
Iron	1.10	0.81–1.50	0.158	0.92	0.77–1.11	0.395	1.04	0.73–1.48	0.846	31.8	< 0.001	0.949
Vitamin												
Folate (vitamin B9)	1.48	0.86–2.54	0.153	1.47	1.01–2.16	0.047	–	–	–	4.4	0.037	0.576
Vitamin B12	0.91	0.72–1.16	0.446	0.90	0.73–1.11	0.337	0.94	0.72–1.23	0.668	28.4	< 0.001	0.183
Vitamin D	0.77	0.65–0.93	0.005	0.83	0.72–0.95	0.006	0.85	0.78–0.94	0.029	17.3	0.004	0.981
Vitamin E	0.75	0.34–1.66	0.481	0.89	0.33–2.38	0.814	–	–	–	2.4	0.293	0.988
Lifestyle factor												
Alcohol drinking	0.73	0.47–1.12	0.149	1.13	0.64–1.99	0.675	0.78	0.53–1.16	0.224	140.0	< 0.001	0.407
Coffee intake	1.00	0.99–1.01	0.969	1.01	0.99–1.02	0.099	1.00	0.99–1.01	0.538	20.0	0.045	0.597
Smoking initiation	1.04	0.91–1.19	0.550	1.12	0.96–1.32	0.146	1.05	0.93–1.19	0.420	469.2	< 0.001	0.774
Physical activity	0.12	0.05–0.32	2 × 10^–5^	0.24	0.07–0.90	0.039	0.15	0.06–0.40	0.012	12.0	0.062	0.066
Serum lipids												
High-density lipoprotein cholesterol	1.14	1.00–1.31	0.057	1.07	0.93–1.22	0.345	1.17	1.06–1.29	0.004	172.0	< 0.001	0.707
Low-density lipoprotein cholesterol	0.95	0.75–1.19	0.651	1.12	0.96–1.31	0.155	0.96	0.86–1.07	0.499	411.6	< 00.01	0.222
Total cholesterol	0.98	0.79–1.23	0.887	1.16	0.99–1.35	0.068	1.00	0.88–1.13	0.951	501.2	< 0.001	0.858
Triglycerides	0.90	0.79–1.02	0.098	0.94	0.81–1.09	0.385	0.88	0.78–1.00	0.052	60.0	0.007	0.808
Inflammatory biomarker												
Tumor necrosis factor	8.83	0.33–237	0.194	5.80	2.06–16.3	0.001	–	–	–	60.7	< 0.001	0.082
C-reactive protein	1.08	0.85–1.38	0.538	0.99	0.84–1.17	0.940	1.08	0.94–1.24	0.262	386.4	< 0.001	0.681
Immunoglobulin E	0.89	0.76–1.05	0.162	0.93	0.78–1.10	0.389	–	–	–	1.6	0.443	0.825
Socioeconomic status												
Education level	0.91	0.78–1.06	0.236	0.93	0.77–1.12	0.430	0.90	0.79–1.02	0.108	1367.8	< 0.001	0.284
Intelligence	1.08	0.91–1.29	0.369	1.04	0.85–1.28	0.707	1.08	0.93–1.25	0.294	365.8	< 0.001	0.392

CI indicates confidence interval; IVW, inverse weighted median; OR, odd ratio. *P* for pleiotropy is the *p* value for the intercept of MR-Egger analysis (*p* < 0.05 indicates the possible pleiotropy)

### Nutrition and lifestyle

Genetically higher serum 25-hydroxyvitamin D levels and physical activity (moderate to vigorous level) were associated with a decreased MS risk in all models and no pleiotropy was detected (Table 2). There was a borderline association between urinary sodium levels and MS in both IVW-random effects and MR-PRESSO models (Table 2). There was no evidence of causal associations of circulating levels of amino acids, fatty acids, or other minerals and vitamins, alcohol drinking, coffee consumption, or smoking with MS risk (Table 2).

### Internal biomarker

Genetic predisposition to higher levels of high-density lipoprotein cholesterol was suggestively associated with a lower risk of MS (Table 2). There was limited evidence supporting causal associations of other serum lipids, tumor necrosis factor, C-reactive protein and immunoglobulin E with MS.

### Systematic review

We obtained 9 meta-analyses on 18 individual risk factors by a systematic search in PubMed. There were limited data from meta-analysis of sun exposure, pesticide-related products exposure, air pollution, exposure to farm animals and pets and antibiotic use in relation to MS. Exposure to organic solvents and Epstein Barr virus infection were positively, whereas cytomegalovirus infection, diphtheria vaccination and tetanus vaccination were inversely associated with MS risk (Supplementary Table 4).

## Discussion

Using MR analysis, we found that 4 out of 65 risk factors were robustly associated with MS risk, including childhood and adulthood body mass index, serum 25-hydroxyvitamin D and physical activity. There was evidence of suggestive associations of type 2 diabetes, waist circumference, body fat percentage, age of puberty and high-density lipoprotein cholesterol with risk of MS. Evidence of latest meta-analyses showed that exposure to organic solvents, Epstein Barr virus and cytomegalovirus virus infection, and diphtheria and tetanus vaccination were associated with MS risk.

Adulthood obesity has been identified as a risk factor for MS in previous studies [3, 12]. The present study confirmed the causal association between high body mass index and an elevated risk of MS using more than ten-fold more SNPs for adulthood body mass index compared with the previous MR study [3]. We additionally assessed the influence of birth weight, childhood body mass index, waist circumstance, body fat percentage, lean body mass, basal metabolic rate, and circulating adiponectin levels on MS. Consistent with observational findings [13], our study observed a causal positive association between childhood obesity and MS risk. Waist circumstance and body fat percentage but not lean body mass showed evidence of possible associations with MS risk, which might shed light on the possible varying effects of obesity phenotypes on MS risk and mechanisms.

Low serum 25-hydroxyvitamin D levels exert detrimental effects on MS development, which has been found in previous studies [2, 14] and verified in the present study. Maternal and neonatal 25-hydroxyvitamin D status has also been found to be associated with MS risk in offspring or later on [15, 16]. We observed a consistent protective effect of moderate to vigorous physical activity on MS risk, which supports observational findings [17]. In addition, increased physical activity level can act as a beneficial rehabilitation strategy for MS patients to manage symptoms, restore function, improve quality of life, and promote wellness [18]. Therefore, from the preventive and therapeutic perspectives, exercise should be promoted among individuals at high risk of MS as well as for MS patients.

Effects of nutritional factors, except vitamin D, on the risk of MS are seldom discussed. Recent prospective cohort studies did not find any associations of potassium, magnesium, calcium and iron with MS risk [19, 20], which is overall consistent with our study. Observational evidence stated a protective effect of omega-3 polyunsaturated fatty acids [21] and a detrimental effect of total polyunsaturated fatty acids [22] on MS risk. Nonetheless, our study examined several individual plasma fatty acids levels and found null associations of these fatty acids with MS. We did not find any causal roles of amino acid and other vitamins in the onset of MS, which are scarcely explored in observational studies.

Observational data showed that the prevalence of both type 1 and type 2 diabetes was higher among MS patients compared with non-MS individuals [23, 24]. The present study revealed a possible association between type 2 diabetes and MS. We found limited evidence supporting a causal effect of type 1 diabetes on MS risk. The reason behind a concurrence between type 1 diabetes and MS in observational studies might be shared genes contributing to susceptibility to both diseases (e.g. *CLEC16A* and *CLECL1*) [25], instead of a causal relationship.

Most studies have detected a decreased MS risk among individuals with postponed puberty age [26, 27], which is consistent with our results. Several population and animal studies have indicated that puberty might influence MS risk or relapse per se or via body mass index and other pathways [28, 29]. Conflicting findings of observational studies have revealed possible roles of cigarette smoking, alcohol drinking, and coffee consumption in the development of MS [12, 30–32]. The present MR study did not confirm a causal influence of those lifestyle factors on MS risk, but we cannot exclude that we may have overlooked weak associations. The causal role of those lifestyle factors on MS risk merit further study if more SNPs are identified for those factors and in studies based on larger number of MS cases and controls.

Among internal biomarkers, previous studies found that serum lipid levels were not associated with MS risk [33]. However, high-density lipoprotein cholesterol was found to play a role in MS fatigue [34]. The present study observed a suggestive positive association between high-density lipoprotein cholesterol and risk of MS. Given inconsistent information on this association, whether high-density lipoprotein cholesterol play a casual role in the development of MS needs more study.

This is the first study to comprehensively investigate the potential risk factors for MS using MR analysis. In addition, for exposures not feasible for MR analysis, a systematic review of the literature was conducted to provide contemporary evidence of risk factors for MS. Evidence from meta-analyses of observational studies can be challenged by potential methodological limitations embedded in such studies. Thus, the findings from meta-analyses need more study. Population bias was largely reduced by using genetic data mainly from individuals with European ancestry. However, findings based on certain analyses using genetic data from multi-ancestries need to be cautiously interpreted and verified. The F-statistic for traits indicated that our results were unlikely biased by weak instruments (F-statistic > 10) [10]. However, the statistical power for some analyses was modest, suggesting that it is likely that some of the null results might suffer from “false negative” findings. Given that MR analysis reflects a lifetime exposure, the obtained effect sizes in the present study might be exaggerated and are not directly comparable with estimates derived from traditional observational studies. All MR analyses assumed linear relationships between the risk factors and MS and no interaction (e.g., the interaction between smoking and human leukocyte antigen genes [35]) or modification effects. We could not assess reverse causality through bidirectional MR analysis because suitable summary-level data were not available for most exposures. Thus, whether there are bidirectional associations between certain exposures and MS needs to be revealed in future study.

## Conclusions

This MR study provides evidence of causal associations of a childhood and adulthood body mass index, serum 25-hydroxyvitamin D and physical activity with MS risk. Our complementary systematic review additionally showed that exposure to organic solvents, Epstein Barr virus and cytomegalovirus virus infection, and diphtheria and tetanus vaccination were associated with MS risk. Taken together, this study suggests that lowering obesity and Epstein Barr virus infection and increasing physical activity and serum vitamin D levels can reduce the risk of MS.

## Electronic supplementary material

Below is the link to the electronic supplementary material.

Supplementary file1 (DOCX 91 kb)
