# Supplementary material for: An atlas on risk factors for multiple sclerosis: a Mendelian randomization study
Source: J Neurol. 2020 Jul 29;268(1):114–24. doi: 10.1007/s00415-020-10119-8 (PMC7815542; doi:10.1007/s00415-020-10119-8)
Supplement: Supplementary file 1 — Supplementary file1 (DOCX 91 kb) [file 415_2020_10119_MOESM1_ESM.docx]

**Supplementary material**

**An atlas on risk factors for multiple sclerosis**

*Shuai Yuan, Ying Xiong, Susanna C. Larsson.*

**Contents**

Supplementary table 1. Selection of potential risk factors for multiple sclerosis

Supplementary table 2. Data sources and instrumental variables used for exposures included in the MR analyses

Supplementary table 3. Selection and information of instrumental variables and power estimation in the present Mendelian randomization study

Supplementary table 4. Information of studies in the discovery stage of International Multiple Sclerosis Consortium (IMSGC)

Supplementary table 5. Associations between risk factors and multiple sclerosis from recent meta-analyses

**Supplementary Table 1**. Selection of potential risk factors for multiple sclerosis

| **Risk factor class** | **Potential risk factor** | **Detail indicator of risk factor** | **Identified from published observational epidemiological studies of MS** | **Included in the present MR analysis** | **Reason for not including in MR analysis *** | **Included in systematic review** |
| --- | --- | --- | --- | --- | --- | --- |
| Health status | Stress | Anxiety disorder | YES | YES |  | NO |
| Health status | Stress | Post-traumatic stress disorder | YES | NO |  | NO |
| Health status | Sleep & Circadian rhythms | Sleep duration | YES | YES |  | NO |
| Health status | Sleep & Circadian rhythms | Short sleep (<7 hours) | YES | YES |  | NO |
| Health status | Sleep & Circadian rhythms | Long sleep (>9 hours) | YES | YES |  | NO |
| Health status | Sleep & Circadian rhythms | Insomnia | YES | YES |  | NO |
| Health status | Sleep & Circadian rhythms | Morningness | YES | YES |  | NO |
| Health status | Sleep & Circadian rhythms | Restless leg syndrome | YES | YES |  | NO |
| Health status | Psychiatric disorder | Major depressive disorder | YES | YES |  | NO |
| Health status | Psychiatric disorder | Obsessive-compulsive disorder | YES | NO | 2 | NO |
| Health status | Psychiatric disorder | Schizophrenia | YES | NO | 2 | NO |
| Health status | Immune disorder | Type 1 diabetes | YES | YES |  | NO |
| Health status | Immune disorder | Latent autoimmune diabetes in adults | NO | YES |  | NO |
| Health status | Immune disorder | Allergic rhinitis | YES | YES |  | NO |
| Health status | Immune disorder | Food allergies | NO | NO |  | NO |
| Health status | Immune disorder | Asthma | YES | YES |  | NO |
| Health status | Immune disorder | Eczema (atopic dermatitis) | YES | YES |  | NO |
| Health status | Immune disorder | Inflammatory bowel disease | YES | NO |  | NO |
| Health status | Immune disorder | Rheumatoid arthritis | YES | YES |  | NO |
| Health status | Immune disorder | Neuromyelitis optica | YES | NO |  | NO |
| Health status | Cardiometabolic factor | Type 2 diabetes | YES | YES |  | NO |
| Health status | Cardiometabolic factor | Fasting glucose | NO | YES |  | NO |
| Health status | Cardiometabolic factor | Fasting insulin | NO | YES |  | NO |
| Health status | Cardiometabolic factor | Hemoglobin A1c | NO | YES |  | NO |
| Health status | Cardiometabolic factor | Hemoglobin | NO | YES |  | NO |
| Health status | Cardiometabolic factor | Systolic blood pressure | YES | YES |  | NO |
| Health status | Cardiometabolic factor | Diastolic blood pressure | YES | YES |  | NO |
| Health status | Cardiometabolic factor | Coronary artery disease | YES | YES |  | NO |
| Health status | Cardiometabolic factor | Peripheral artery disease | YES | YES |  | NO |
| Health status | Obesity-related trait | Birth weight | YES | YES |  | NO |
| Health status | Obesity-related trait | Childhood body mass index | YES | YES |  | NO |
| Health status | Obesity-related trait | Adulthood body mass index | YES | YES |  | NO |
| Health status | Obesity-related trait | Waist circumference | NO | YES |  | NO |
| Health status | Obesity-related trait | Lean body mass | NO | YES |  | NO |
| Health status | Obesity-related trait | Body fat percentage | NO | YES |  | NO |
| Health status | Obesity-related trait | Circulating adiponectin | NO | YES |  | NO |
| Health status | Obesity-related trait | Body mass index change | YES | NO | 1 | NO |
| Health status | Hormone-related factor | Age at puberty | YES | YES |  | NO |
| Health status | Hormone-related factor | Age of menopause | NO | YES |  | NO |
| Health status | Hormone-related factor | Testosterone | YES | NO |  | NO |
| Health status | Hormone-related factor | Sex-hormone binding globulin | YES | NO |  | NO |
| Health status | Other factors | Apoptosis | YES | NO | 1 | NO |
| Health status | Other factors | Brain atrophy | YES | NO | 1 | NO |
| Health status | Other factors | Concussion in adolescence | YES | NO | 1 | NO |
| Health status | Other factors | Cerebrospinal fluid (CSF) CXCL13 | YES | NO | 1 | NO |
| Health status | Other factors | Estimated bone mineral density | YES | YES |  | NO |
| Health status | Other factors | Family history of chronic diseases | YES | NO | 1 | NO |
| Health status | Other factors | Gut microbiome | YES | NO | 1 | NO |
| Health status | Other factors | Melatonin | YES | NO | 1 | NO |
| Health status | Other factors | Migraine | YES | YES |  | NO |
| Health status | Other factors | Migraine without aura | YES | YES |  | NO |
| Health status | Other factors | Mitochondrial dysfunction | YES | NO | 1 | NO |
| Health status | Other factors | Neurofilament light chain | YES | NO | 1 | NO |
| Health status | Other factors | Prematurity | YES | NO | 1 | NO |
| Health status | Other factors | Septicemia | YES | NO | 1 | NO |
| Health status | Other factors | Systemic sclerosis | YES | NO | 1 | NO |
| Health status | Other factors | Spinal cord injury | YES | NO | 1 | NO |
| Health status | Other factors | Urinary complications | YES | NO | 1 | NO |
| Health status | Other factors | Uric acid | YES | YES |  | NO |
| Nutritional factor | Amino acid | Carnitine | NO | YES |  | NO |
| Nutritional factor | Amino acid | Homocysteine | YES | YES |  | NO |
| Nutritional factor | Amino acid | Isoleucine | NO | NO |  | NO |
| Nutritional factor | Amino acid | Leucine | NO | NO |  | NO |
| Nutritional factor | Amino acid | Valine | NO | NO |  | NO |
| Nutritional factor | Fatty acid | α-Linolenic acid | YES | NO |  | NO |
| Nutritional factor | Fatty acid | Eicosapentaenoic acid | YES | NO |  | NO |
| Nutritional factor | Fatty acid | Docosahexaenoic acid | YES | NO |  | NO |
| Nutritional factor | Fatty acid | Docosapentaenoic acid | YES | YES |  | NO |
| Nutritional factor | Fatty acid | Linoleic acid | YES | NO |  | NO |
| Nutritional factor | Fatty acid | Arachidonic acid | YES | NO |  | NO |
| Nutritional factor | Fatty acid | Palmitoleic acid | YES | YES |  | NO |
| Nutritional factor | Fatty acid | Oleic acid | YES | NO |  | NO |
| Nutritional factor | Fatty acid | Palmitic acid | YES | NO |  | NO |
| Nutritional factor | Fatty acid | Stearic acid | YES | YES |  | NO |
| Nutritional factor | Mineral | Calcium | NO | YES |  | NO |
| Nutritional factor | Mineral | Magnesium | NO | YES |  | NO |
| Nutritional factor | Mineral | Sodium | YES | YES |  | NO |
| Nutritional factor | Mineral | Potassium | NO | YES |  | NO |
| Nutritional factor | Mineral | Iron | YES | YES |  | NO |
| Nutritional factor | Mineral | Selenium | NO | YES |  | NO |
| Nutritional factor | Vitamin | Folate (vitamin B9) | YES | YES |  | NO |
| Nutritional factor | Vitamin | Vitamin B12 | YES | YES |  | NO |
| Nutritional factor | Vitamin | Vitamin C | NO | YES |  | NO |
| Nutritional factor | Vitamin | Vitamin D | YES | YES |  | NO |
| Nutritional factor | Vitamin | Maternal vitamin D | YES | NO | 1 | NO |
| Nutritional factor | Vitamin | Vitamin E | NO | YES |  | NO |
| Nutritional factor | Diet | Dairy intake | YES | NO | 2 | NO |
| Nutritional factor | Diet | Dietary inflammatory index | YES | NO | 1 | NO |
| Nutritional factor | Diet | Dietary pattern | YES | NO | 1 | NO |
| Nutritional factor | Diet | Flavonoids | YES | NO | 1 | NO |
| Nutritional factor | Diet | Fish intake | YES | NO | 1 | NO |
| Nutritional factor | Diet | Fruit intake | YES | NO | 1 | NO |
| Nutritional factor | Diet | Mealtime | YES | NO | 1 | NO |
| Nutritional factor | Diet | Seafood consumption | YES | NO | 1 | NO |
| Lifestyle | Lifestyle factor | Cigarette smoking | YES | YES |  | NO |
| Lifestyle | Lifestyle factor | Waterpipe smoking | YES | NO | 1 | NO |
| Lifestyle | Lifestyle factor | Nicotine exposure | YES | NO | 1 | NO |
| Lifestyle | Lifestyle factor | Alcohol drinking | YES | YES |  | NO |
| Lifestyle | Lifestyle factor | Coffee consumption | YES | YES |  | NO |
| Lifestyle | Lifestyle factor | Physical activity | YES | YES |  | NO |
| Internal biomarker | Lipids | High-density lipoprotein cholesterol | YES | YES |  | NO |
| Internal biomarker | Lipids | Low-density lipoprotein cholesterol | YES | YES |  | NO |
| Internal biomarker | Lipids | Total cholesterol | YES | YES |  | NO |
| Internal biomarker | Lipids | Total triglyceride | YES | YES |  | NO |
| Internal biomarker | Inflammatory biomarker | Tumor necrosis factor | YES | YES |  | NO |
| Internal biomarker | Inflammatory biomarker | Interleukin 2 | YES | NO |  | NO |
| Internal biomarker | Inflammatory biomarker | Interleukin 7 | YES | NO | 2 | NO |
| Internal biomarker | Inflammatory biomarker | Interleukin 10 | YES | NO | 3 | NO |
| Internal biomarker | Inflammatory biomarker | Interleukin 17 | YES | NO |  | NO |
| Internal biomarker | Inflammatory biomarker | Interleukin 18 | YES | NO |  | NO |
| Internal biomarker | Inflammatory biomarker | C-reactive protein | NO | YES |  | NO |
| Internal biomarker | Inflammatory biomarker | Immunoglobulin E | NO | YES |  | NO |
| Internal biomarker | Immune cell | Lymphocyte | YES | NO |  | NO |
| Internal biomarker | Immune cell | Anti-α1,3-Gal IgG | YES | NO | 1 | NO |
| Demographic factor | Socioeconomic status | Education level | YES | YES |  | NO |
| Demographic factor | Socioeconomic status | Intelligence | YES | YES |  | NO |
| Demographic factor | Socioeconomic status | Shift work | YES | NO | 1 | NO |
| Demographic factor | Socioeconomic status | Professional automobile drivers | YES | NO | 1 | NO |
| Other factors | Other factors | Sun exposure | YES | NO | 1 | NO |
| Other factors | Other factors | High latitude | YES | NO | 1 | YES |
| Other factors | Other factors | Season of birth | YES | NO | 1 | YES |
| Other factors | Other factors | Solar and geomagnetic activity | YES | NO | 1 | YES |
| Other factors | Other factors | Pesticide-related products | YES | NO | 1 | NO |
| Other factors | Other factors | Air pollution | YES | NO | 1 | NO |
| Other factors | Other factors | Exposure to organic solvents | YES | NO | 1 | YES |
| Other factors | Other factors | Farm animals and pets | YES | NO | 1 | NO |
| Infection | Infection | EBV infection | YES | NO | 1 | YES |
| Infection | Infection | ERV reactivation | YES | NO | 1 | YES |
| Infection | Infection | Cytomegalovirus infection | YES | NO | 1 | YES |
| Infection | Infection | Vaccines | YES | NO | 1 | YES |
| Infection | Infection | Toxoplasma gondii infection | YES | NO | 1 | YES |
| Infection | Infection | Antibiotic use | YES | NO | 1 | NO |

Risk factor detection systematic review strategy: (("Risk Factors"[Mesh] or Risk Factor[tiab])) AND (("Multiple Sclerosis"[Mesh] or Disseminated Sclerosis[ti] or MS[tiab] or Multiple Sclerosis[ti]) AND "last 5 years"[PDat] AND Humans[Mesh]). In total, 1863 articles have been identified and 91 general possible risk factors have been proposed after title and abstract screening.

* Reasons for not including risk factor in MR analysis:1: Trait-associated SNPs not available for use as IVs; 2: There was no single SNP reporting in studies reaching 5×10^-8^; 3: SNPs cannot be used due to significant population difference.

**Supplementary table 2.** Data sources and instrumental variables used for exposures included in the MR analyses

| **Exposure** | **Cases or sample size** | **Controls** | **Population** | **SNPs^c^** | **Variance (%)^d^** | **PubMed ID** |
| --- | --- | --- | --- | --- | --- | --- |
| **Psychiatric factor** |  |  |  |  |  |  |
| Lifetime anxiety disorder | 25 453 | 58 113 | European | 5 | 0.5 ^e^ | BioRxiv |
| Major depressive disorder | 414 055 | 892 299 | European | 95 | 1.8 ^d^ | 30718901 |
| Sleep duration | 446 118 | NA | European | 76 | 0.7 ^e^ | 30846698 |
| Short sleep (<7 hours) | 106 192 | 305 742 | European | 26 | 1.2 ^d^ | 30846698 |
| Long sleep (>9 hours) | 34 184 | 305 742 | European | 6 | 0.7 ^d^ | 30846698 |
| Insomnia | 397 972 | 933 038 | European | 238 | 2.6 ^e^ | 30804565 |
| Morningness | 372 765 | 278 530 | European | 329 | 11.9 ^d^ | 30696823 |
| Restless leg syndrome | 15 126 | 95 725 | European | 20 | 11.7 ^e^ | 29029846 |
| **Autoimmune disorder** |  |  |  |  |  |  |
| Type 1 diabetes | 9934 | 16 956 | European | 18 | 6.7 ^e^ | 21980299 |
| Latent autoimmune diabetes in adults | 2634 | 5947 | European | 3 | 0.7 ^d^ | 30254083 |
| Allergic rhinitis | 59 762 | 152 358 | European | 33 | 4.7 ^d^ | 30013184 |
| Asthma | 10 549 | 47 146 | European | 18 | 13.7 ^d^ | 30552067 |
| Eczema (atopic dermatitis) | 18 900 | 84 166 | European | 20 | 7.2 ^d^ | 26482879 |
| Rheumatoid arthritis | 18 136 | 49 724 | European | 27 | 8.1 ^d^ | 24390342 |
| **Cardiometabolic factor** |  |  |  |  |  |  |
| Type 2 diabetes | 74 124 | 824 006 | European | 179 | 1.0 ^d^ | 30297969 |
| Fasting glucose ^a^ | 133 010 | NA | European | 35 | 4.8 ^d^ | 22885924 |
| Fasting insulin ^a^ | 133 010 | NA | European | 18 | 1.0 ^d^ | 22885924 |
| Hemoglobin A1c ^a^ | up to 159 940 | NA | Mixed | 48 | 4.7 ^e^ | 28898252 |
| Hemoglobin ^a^ | 135 367 | NA | Mixed | 25 | NA | 23222517 |
| Diastolic blood pressure | >1 million | NA | Mixed | 271 | 5.3 ^e^ | 30224653 |
| Systolic blood pressure | >1 million | NA | Mixed | 229 | 5.7 ^e^ | 30224653 |
| Coronary artery disease | 60 801 | 123 504 | Mixed | 45 | 1.7 ^e^ | 26343387 |
| Peripheral artery disease | 36 424 | 601 044 | European | 18 | 3.5 ^d^ | 31285632 |
| **Obesity-related factor** |  |  |  |  |  |  |
| Birth weight | up to 500 000 | NA | European | 92 | 2.5 ^d^ | 30305743 |
| Childhood body mass index | 35 668 | NA | Mixed | 15 | 2.2 ^d^ | 26604143 |
| Adulthood body mass index | 250 000 | NA | European | 963 | 7.8 ^d^ | 30124842 |
| Waist circumference | 500 000 | NA | European | 314 | 4.6 ^d^ | 30305743 |
| Lean body mass | 47 227 | NA | European | 7 | 4.6 ^d^ | 30721968 |
| Body fat percentage | up to 500 000 | NA | European | 364 | 5.3 ^d^ | 30305743 |
| Basal metabolic rate | up to 500 000 | NA | European | 675 | 11.8 ^d^ | 30305743 |
| Circulating adiponectin ^a^ | 45 891 | NA | Mixed | 10 | NA | 22479202 |
| **Hormone-related factor** |  |  |  |  |  |  |
| Age of puberty | up to 370 000 | NA | Mixed | 339 | 7.4 ^e^ | 28436984 |
| Age of natural menopause | up to 70 000 | NA | European | 42 | NA | 26414677 |
| **Other factors** |  |  |  |  |  |  |
| Migraine | 59 674 | 316 078 | European | 33 | 4.4 ^d^ | 27322543 |
| Migraine without aura | 2326 | 4580 | European | 6 | 7.8 ^d^ | 22683712 |
| Estimated bone mineral density | 426 824 | NA | European | 993 | 20.7 ^e^ | 30598549 |
| Uric acid ^a^ | 288 649 | NA | European | 123 | 5.3 ^e^ | 31578528 |
| **Amino acid** |  |  |  |  |  |  |
| Carnitine ^a^ | 7824 | NA | European | 18 | 13.8 ^d^ | 24816252 |
| Homocysteine ^a^ | 44 147 | NA | European | 16 | 3.3 ^d^ | 23824729 |
| Isoleucine ^a^ | 16 596 | NA | European | 4 | 1.1 ^d^ | 27898682 |
| **Plasma** **fatty acid** |  |  |  |  |  |  |
| Docosapentaenoic acid | 8866 | NA | European | 3 | 3.2 ^d^ | 21829377 |
| Linoleic acid | 8631 | NA | European | 3 | 2.1 ^d^ | 24823311 |
| Palmitoleic acid | 8961 | NA | European | 5 | 3.5 ^d^ | 23362303 |
| Stearic acid | 8964 | NA | European | 3 | 2.1 ^d^ | 23362303 |
| **Mineral** |  |  |  |  |  |  |
| Calcium ^a^ | 39 400 | NA | European | 7 | 0.9 ^d^ | 24068962 |
| Magnesium ^a^ | 15 366 | NA | European | 6 | 1.6 ^d^ | 20700443 |
| Sodium ^b^ | 446 237 | NA | European | 47 | NA | 31409800 |
| Potassium ^b^ | 446 238 | NA | European | 12 | NA | 31409800 |
| Iron ^a^ | 48 972 | NA | European | 5 | 3.4 ^d^ | 25352340 |
| **Vitamin** |  |  |  |  |  |  |
| Folate (vitamin B9) ^a^ | 37 341 | NA | Mixed | 3 | 0.8 ^d^ | 23754956 |
| Vitamin B12 ^a^ | 45 576 | NA | Mixed | 10 | 4.5 ^d^ | 23754956 |
| Vitamin D ^a^ | 121 640 | NA | European | 7 | 5.3 ^d^ | 29343764 |
| Vitamin E ^a^ | 5006 | NA | European | 3 | 1.7 ^e^ | 21729881 |
| **Lifestyle factor** |  |  |  |  |  |  |
| Alcohol drinking | 941 280 | NA | European | 87 | 2.5 ^e^ | 30643251 |
| Coffee consumption | 375 833 | NA | European | 15 | 0.5 ^e^ | 31046077 |
| Cigarettes smoked per day | 337 334 | NA | European | 45 | 4.0 ^e^ | 30643251 |
| Smoking initiation | 1 232 091 | NA | European | 349 | 1.0 ^e^ | 30643251 |
| Physical activity | 377 234 | NA | European | 6 | 0.8 ^d^ | 29899525 |
| **Serum lipids** |  |  |  |  |  |  |
| High-density lipoprotein cholesterol ^a^ | 188 577 | NA | Mixed | 68 | 1.6 ^d^ | 24097068 |
| Low-density lipoprotein cholesterol ^a^ | 188 577 | NA | Mixed | 58 | 2.4 ^d^ | 24097068 |
| Total cholesterol ^a^ | 188 577 | NA | Mixed | 74 | 2.6 ^d^ | 24097068 |
| Triglycerides ^a^ | 188 577 | NA | Mixed | 37 | 2.1 ^d^ | 24097068 |
| **Inflammatory biomarker** |  |  |  |  |  |  |
| Tumor necrosis factor ^a^ | 30 912 | NA | European | 3 | 0.6 ^e^ | - |
| C-reactive protein ^a^ | 204 402 | NA | European | 55 | 7.0 ^e^ | 30388399 |
| Immunoglobulin E ^a^ | 6819 | NA | European | 3 | 1.6 ^e^ | 22075330 |
| **Socioeconomic status** |  |  |  |  |  |  |
| Educational level | 1 131 881 | NA | European | 1197 | 12.0 ^d^ | 30038396 |
| Intelligence | 269 867 | NA | European | 230 | 5.2 ^d^ | 29942086 |

NA indicates not available; SNP, single nucleotide polymorphism. Variables without controls information are continuous variables.

^a^ Measurement of these indicators was based on serum levels. ^b^ Measurement of these indicators was based on urinary levels.

^c^ Numbers of SNPs used in the present Mendelian randomization analyses.

^d^ Variance estimation was based on the formula 𝑅^2^=2×MAF×(1−MAF)×(beta/SD)^2^ (MAF indicates minor allele frequency; beta estimation was based on MAF; and SD was one) for continuous traits with SD unit or binary traits without variance information in original genome-wide association studies.

^e^ For continuous traits that were not scaled into SD unit, variance explained was extracted from the original genome-wide association studies. For binary traits with variance information in original genome-wide association studies, variance was extracted from the paper directly.

**Supplementary table 3**. Selection and information of instrumental variables and power estimation in the present Mendelian randomization study

| **Exposure** | **SNPs in GWAS** | **Not available in MS dataset** | **Unit** | **Detectable OR* at 80% power** |
| --- | --- | --- | --- | --- |
| **Psychiatric factor** |  |  |  |  |
| Lifetime anxiety disorder | 5 | 0 | - | ≤0.83 or ≥1.19 |
| Major depressive disorder | 102 | 0 | - | ≤0.80 or ≥1.24 |
| Sleep duration | 78 | 2 | Hours/day | ≤0.69 or ≥1.40 |
| Short sleep (<7 hours) | 27 | 1 | - | ≤0.75 or ≥1.30 |
| Long sleep (>9 hours) | 8 | 0 | - | ≤0.69 or ≥1.40 |
| Insomnia | 248 | 5 | - | ≤0.91 or ≥1.09 |
| Morningness | 351 | 6 | - | ≤0.91 or ≥1.09 |
| Restless leg syndrome | 20 | 0 | - | ≤0.91 or ≥1.09 |
| **Autoimmune disorder** |  |  |  |  |
| Type 1 diabetes | 19 | 0 | - | ≤0.89 or ≥1.12 |
| Latent autoimmune diabetes in adults | 4 | 0 | - | ≤0.69 or ≥1.40 |
| Allergic rhinitis | 41 | 7 | - | ≤0.87 or ≥1.15 |
| Asthma | 24 | 6 | - | ≤0.92 or ≥1.09 |
| Eczema (atopic dermatitis) | 21 | 1 | - | ≤0.89 or ≥1.12 |
| Rheumatoid arthritis | 27 | 0 | - | ≤0.90 or ≥1.11 |
| **Cardiometabolic factor** |  |  |  |  |
| Type 2 diabetes | 190 | 6 | - | ≤0.73 or ≥1.33 |
| Fasting glucose ^a^ | 35 | 0 | SD | ≤0.87 or ≥1.15 |
| Fasting insulin ^a^ | 18 | 0 | SD | ≤0.73 or ≥1.33 |
| Hemoglobin A1c ^a^ | 50 | 1 | % unit increase | ≤0.87 or ≥1.15 |
| Hemoglobin ^a^ | 27 | 0 | g/dL | - |
| Diastolic blood pressure | 280 | 3 | 20-mm Hg increase | ≤0.87 or ≥1.14 |
| Systolic blood pressure | 237 | 3 | 20-mm Hg increase | ≤0.88 or ≥1.13 |
| Coronary artery disease | 46 | 1 | - | ≤0.79 or ≥1.25 |
| Peripheral artery disease | 19 | 1 | - | ≤0.85 or ≥1.17 |
| **Obesity-related factor** |  |  |  |  |
| Birth weight | 93 | 1 | SD | ≤0.82 or ≥1.20 |
| Childhood body mass index | 15 | 0 | SD | ≤0.81 or ≥1.22 |
| Adulthood body mass index | 964 | 1 | SD | ≤0.89 or ≥1.11 |
| Waist circumference | 319 | 5 | SD | ≤0.87 or ≥1.15 |
| Lean body mass | 7 | 0 | SD | ≤0.87 or ≥1.15 |
| Body fat percentage | 370 | 6 | SD | ≤0.87 or ≥1.14 |
| Basal metabolic rate | 693 | 18 | SD | ≤0.91 or ≥1.09 |
| Circulating adiponectin ^a^ | 10 | 0 | µg/mL | - |
| **Hormone-related factor** |  |  |  |  |
| Age of puberty | 389 | 42 | Year | ≤0.89 or ≥1.12 |
| Age of natural menopause | 42 | 0 | Year | - |
| **Other factors** |  |  |  |  |
| Migraine | 38 | 1 | - | ≤0.86 or ≥1.15 |
| Migraine without aura | 6 | 0 | - | ≤0.89 or ≥1.11 |
| Estimated bone mineral density | 1103 | 70 | g/cm^2^ | ≤0.93 or ≥1.07 |
| Uric acid ^a^ | 123 | 0 | mg/dL | ≤0.87 or ≥1.14 |
| **Amino acid** |  |  |  |  |
| Carnitine ^a^ | 18 | 0 | SD | ≤0.92 or ≥1.09 |
| Homocysteine ^a^ | 18 | 0 | SD | ≤0.84 or ≥1.18 |
| Isoleucine ^a^ | 4 | 0 | SD | ≤0.73 or ≥1.33 |
| **Fatty acid** |  |  |  |  |
| Docosapentaenoic acid ^a^ | 3 | 0 | SD | ≤0.84 or ≥1.18 |
| Linoleic acid ^a^ | 3 | 0 | SD | ≤0.81 or ≥1.22 |
| Palmitoleic acid ^a^ | 5 | 0 | SD | ≤0.85 or ≥1.17 |
| Stearic acid ^a^ | 3 | 0 | SD | ≤0.81 or ≥1.22 |
| **Mineral** |  |  |  |  |
| Calcium ^a^ | 7 | 0 | SD | ≤0.72 or ≥1.35 |
| Magnesium ^a^ | 6 | 0 | SD | ≤0.78 or ≥1.26 |
| Sodium ^b^ | 50 | 0 | mmol/L | - |
| Potassium ^b^ | 13 | 0 | mmol/L | - |
| Iron ^a^ | 5 | 0 | SD | ≤0.85 or ≥1.17 |
| **Vitamin** |  |  |  |  |
| Folate (vitamin B9) ^a^ | 3 | 0 | SD | ≤0.71 or ≥1.37 |
| Vitamin B12 ^a^ | 15 | 5 | SD | ≤0.86 or ≥1.15 |
| Vitamin D ^a^ | 7 | 0 | SD | ≤0.87 or ≥1.14 |
| Vitamin E ^a^ | 3 | 0 | Natural log of mg/L | ≤0.79 or ≥1.25 |
| **Lifestyle factor** |  |  |  |  |
| Alcohol drinking | 99 | 6 | Drinks/week | ≤0.82 or ≥1.20 |
| Coffee consumption | 15 | 0 | 50% increase in coffee consumption | ≤0.64 or ≥1.48 |
| Cigarettes smoking per day | 55 | 0 | Cigarettes/day | ≤0.86 or ≥1.16 |
| Smoking initiation | 378 | 15 | - | ≤0.73 or ≥1.33 |
| Physical activity | 9 | 0 | SD | ≤0.71 or ≥1.37 |
| **Serum lipids** |  |  |  |  |
| High-density lipoprotein cholesterol ^a^ | 71 | 2 | SD | ≤0.78 or ≥1.26 |
| Low-density lipoprotein cholesterol ^a^ | 58 | 0 | SD | ≤0.82 or ≥1.21 |
| Total cholesterol ^a^ | 74 | 0 | SD | ≤0.83 or ≥1.20 |
| Total triglycerides ^a^ | 40 | 1 | SD | ≤0.81 or ≥1.22 |
| **Inflammatory biomarker** |  |  |  |  |
| Tumor necrosis factor ^a^ | 4 | 0 | Natural log-transformed levels in mg/L | ≤0.85 or ≥1.17 |
| C-reactive protein ^a^ | 58 | 0 | Natural log-transformed levels in mg/L | ≤0.89 or ≥1.12 |
| Immunoglobulin E ^a^ | 3 | 0 | Natural log-transformed levels in mg/L | ≤0.78 or ≥1.26 |
| **Socioeconomic status** |  |  |  |  |
| Educational level | 1271 | 21 | SD | ≤0.91 or ≥1.09 |
| Intelligence | 242 | 3 | SD | ≤0.87 or ≥1.14 |

LD indicates linkage disequilibrium; NA, not available; PMID, PubMed ID; SNP, single nucleotide polymorphism.

^a^ Measurement of these indicators was based on serum levels. ^b^ Measurement of these indicators was based on urinary levels. ^c^ The European samples from the 1000 genomes project are used to estimate linkage disequilibrium between SNPs [1]. ^d^ F calculation used the formula: F=((n-k-1)/k)×(R^2^/(1-R^2^)) [n, sample size; k, numbers of used SNPs; R^2^, explained variance by used SNPs] [2]. ^*^ Power calculation were based on the online application “mRnd: Power calculations for Mendelian Randomization” (<http://cnsgenomics.com/shiny/mRnd/>) [3]. There were 14498 multiple sclerosis cases and 24091 controls in the discovery stage of International Multiple Sclerosis Consortium. Thus, the sample size was 38589 and the proportion of cases was 38%.

**Supplementary table 4**. Information of studies in the discovery stage of International Multiple Sclerosis Consortium (IMSGC)[4]

| **Area** | **Cases recruitment** | **Cases** | **Controls** | **F:M** | **AAE (years)** | **AAO (years)** | **EDSS** | **MSSS** | **PPMS (%)** | **SNPs** |
| --- | --- | --- | --- | --- | --- | --- | --- | --- | --- | --- |
| ANZ | All cases were self-identified volunteers recruited at centres located in Adelaide, Brisbane, Gold Coast, Hobart, Melbourne, Newcastle, Perth, Sydney and in New Zealand. All were confirmed by Neurologists. | 247 | 944 | 4.04 | 56.7 | 35.1 | 4.5 | 4.6 | 8.3 | 147722 |
| Belgium | Samples were collected under coordination of the Neurology Department of the University Hospitals Leuven amongst out-patients and hospitalized patients with definite multiple sclerosis attending the Neurology Department of the University Hospitals Leuven or the “National MS Center” in Melsbroek. | 302 | 1703 | 1.65 | 48.5 | 34.4 | 4.2 | 5 | 12.3 | 150708 |
| Denmark | Patients were recruited between 1996-2009 by neurologists at multiple sclerosis centers from across all of Denmark, although most patients originate from the Copenhagen area. | 741 | 835 | 2.21 | 43.4 | 31.2 | 3.6 | 4.5 | 2.7 | 144726 |
| Finland | Cases were recruited from seven centres (Helsinki University Central Hospital, Tampere University Hospital, Kuopio University hospital, Oulu University Hospital, Seinäjoki Central Hospital, Satakunta Central Hospital and Rovaniemi Central Hospital) across Finland. All were identified in hospital clinics by experienced neurologists. | 221 | 486 | 2.35 | 43.4 | 31.1 | 4.4 | 5.2 | 11.3 | 141359 |
| France | The French MS Genetics group (REFGENSEP) has been prospectively collecting samples with twenty-three centers covering France. Some patients volunteered in response to advertising campaigns through patient associations. | 386 | 354 | 2.54 | 45.4 | 32.4 | 3.9 | 4.8 | 13.4 | 146244 |
| Germany | Hamburg: Samples were collected from patients regularly seen in the MS outpatient clinic and day hospital of the Institute for Neuroimmunology and Clinical MS Research.  Munich: Samples from the TU in Munich can be stratified in three cohorts of cases and one cohort of controls.  University Mainz: Samples were collected from outpatient clinics. Patients originate from different regions in Germany with the vast majority being of self-reported Caucasian descent. Collection was performed in Outpatient Clinics. | 2582 | 5545 | 2.57 | 40.5 | 32.6 | 2.9 | 3.8 | 5 | 153595 |
| Italy | Piedmont: Patients were collected from continental Italy (excluding Sardinia) as part of the PROGEMUS (PROgnostic GEnetic factors in MUltiple Sclerosis) project. These patients were all recruited from hospital-based clinics and the mean participation rate was approximately 60% (range 20%-90%)  San Raffaele, Milan: Patients were recruited between 2000 and 2010 by multiple sclerosis neurologists at the Ospedale San Raffaele (OSR) in Milan. They are outpatients and hospitalized patients from continental Italy, with a higher proportion from northern Italy. | 957 | 1255 | 1.96 | 41.3 | 30.4 | 2.6 | 3 | 7 | 153086 |
| Norway | The Norwegian multiple sclerosis samples were derived from two sources; the Oslo MS DNA biobank and the Norwegian Multiple Sclerosis Registry and Biobank held in Bergen. | 894 | 674 | 2.66 | 48.7 | 33.2 | 4.5 | 4.9 | 15.8 | 147413 |
| Sweden | The Swedish cases and controls were recruited from four different investigations: The Epidemiological Investigation of Multiple Sclerosis, Genetic Environment study in Multiple Sclerosis, nation-wide study of natalizumab treatment in multiple sclerosis patients, samples collected during routine neurological diagnostic work-up at Karolinska University Hosptial and Danderyd Hospital (Stockholm, Sweden) and 721 blood donors. | 2153 | 2331 | 2.64 | 45 | 35.4 | 3.3 | 4.7 | 5.5 | 150124 |
| UK | The majority of UK cases were collected through a national recruitment project (“the genetic analysis of multiple sclerosis”) coordinated by the Department of Clinical Neurosciences at the University of Cambridge and involving additional recruitment centres based in UK cities - Aberdeen, Birmingham, Bristol, Cardiff, Exeter, Hull, Ipswich, Leicester, London, Manchester, Newcastle, Norwich, Nottingham, Oxford, Peterborough, Preston, Plymouth, Poole, Rotherham, Sheffield, Southampton and Stoke. These cases were supplemented by additional cases recruited as part of local natural history studies in the South West of England28 and South Wales,29 together with samples recruited as part of the Northern Isles Multiple Sclerosis (NIMS) study 30 and samples from the UK multiple sclerosis tissue bank. | 4324 | 4422 | 2.84 | 48 | 33.4 | 4.3 | 5.2 | 10.3 | 152151 |
| USA | Brigham & Women’s Hospital (BWH), Boston MA: Study participants were recruited through the Partners MS Center in Boston, MA. All samples were collected at the MS Center and processed on site to extract DNA.  University of Miami (UM): Study participants were recruited using multiple ascertainment approaches. However, the majority of participants were enrolled through the University of Miami Health System’s designated MS Center of Excellence. Additional participants were recruited via multiple sclerosis community outreach events and support group meetings.  University of California San Francisco (UCSF): Study participants were recruited from the UCSF MS clinic and from other collaborating sites across the United States using common inclusion and exclusion criteria.  Vanderbilt University (Nashville): All samples were obtained from the Vanderbilt DNA Biorepository (BioVU). This repository connects a de-identified version of Vanderbilt’s electronic medical records to DNA samples extracted from waste blood obtained from the phlebotomy labs at Vanderbilt. Thus, all samples were obtained through the Vanderbilt Hospitals and Clinics and represent a primary catchment area of Middle Tennessee, U.S.A. Cases were defined using algorithms focused on ICD-9 billing codes, prescribed multiple sclerosis treatments, and keywords located in the text. | 1691 | 5542 | 3.68 | 52.3 | 33.7 | 4.4 | 4.2 | 6.7 | 155555 |
| Total | All of above | 14498 | 24091 | 2.69 | 45.3 | 33.1 | 3.7 | 4.6 | 8.2 | 161311* |

ANZ indicates Australia and New Zealand; UK, United Kingdom, USA, United States of America, F:M, Female to male ratio, AAE, Age at examination (in years), AAO, Age at onset (in years), EDSS, Expanded Disability Status Score, MSSS, Multiple Sclerosis Severity Score, PPMS, Primary progressive multiple sclerosis (% of patients with disease course information that have PPMS); SNPs, single nucleotide polymorphisms.

* Includes 7688 SNPs from the MHC region, 104425 SNPs from the 184 fine mapping regions and 49198 from elsewhere in the genome.**Supplementary table 5**. Associations between risk factors and multiple sclerosis from recent meta-analyses

| **Exposure** | **PubMed ID** | **Published year** | **Included studies** | **Cases** | **Controls** | **OR** | **95% CI** | **P** | **I^2^ (%)** |
| --- | --- | --- | --- | --- | --- | --- | --- | --- | --- |
| **Other factors** |  |  |  |  |  |  |  |  |  |
| Latitude [5] | 31217172 | 2019 | 94 | NA | NA | Prevalence increased 5.27/100 000 per degree latitude | | | |
| Solar and geomagnetic activity [6] | 25295148 | 2019 | 2 | NA | NA | Incidence was correlated with solar wind velocity and planetary A index | | | |
| Season of birth^*^ [7] | 31055633 | 2019 | 22 | 145 672 | 7 5169 550 | 1.08 | 1.03-1.14 | NA | 90 |
| Exposure to organic solvents [8] | 23284705 | 2012 | 15 | 1811 | 1 140 990 | 1.54 | 1.03-2.29 | 0.030 | 77 |
| **Infection** |  |  |  |  |  |  |  |  |  |
| Anti-EBNA IgG [9] | 23585874 | 2013 | 30 | 3347 | 3295 | 4.47 | 3.26-6.11 | <0.001 | 43 |
| Anti-VCA IgG [9] | 23585874 | 2013 | 24 | 2839 | 3026 | 4.51 | 2.84-7.16 | <0.001 | 59 |
| Anti-EA IgG [9] | 23585874 | 2013 | 14 | 518 | 501 | 1.4 | 0.9-2.1 | 0.090 | 70 |
| Anti-EBV IgG sero-negativity [9] | 23585874 | 2013 | 7 | 20 | 170 | 0.13 | 0.05-0.33 | <0.001 | 56 |
| Cytomegalovirus infection [10] | 23999606 | 2014 | 11 | NA | NA | 0.77 | 0.67-0.87 |  |  |
| **Vaccination** |  |  |  |  |  |  |  |  |  |
| HBV vaccination [11] | 30260264 | 2018 | 7 | 15 389 | 11 856 | 1.19 | 0.96-1.49 | NA | NA |
| BCG vaccination [12] | 21431896 | 2011 | 6 | 536 | 751 | 0.96 | 0.69-1.34 | 0.820 | 0 |
| Diphtheria vaccination [12] | 21431896 | 2011 | 3 | 237 | 387 | 0.6 | 0.4-0.91 | 0.020 | 0 |
| Influenza vaccination [12] | 21431896 | 2011 | 4 | 14 997 | 10 128 | 0.97 | 0.77-1.23 | 0.810 | 37 |
| MMR vaccination [12] | 21431896 | 2011 | 3 | 568 | 1880 | 1.02 | 0.64-1.62 | 0.940 | 0 |
| Polio vaccination [12] | 21431896 | 2011 | 7 | 570 | 725 | 0.87 | 0.61-1.26 | 0.460 | 29 |
| Tetanus vaccination [12] | 21431896 | 2011 | 8 | 923 | 3203 | 0.71 | 0.57-0.88 | 0.002 | 17 |
| Typhoid fever vaccination [12] | 21431896 | 2011 | 4 | 288 | 467 | 1.05 | 0.72-1.53 | 0.810 | 14 |
| Toxoplasma gondii infection [13] | 29954625 | 2018 | 5 | 669 | 770 | 0.72 | 0.49-1.06 | 0.096 | 59 |

CI indicates confidence interval; EA, early antigen; EBNA, Epstein–Barr virus nuclear antigen; EBV, Epstein Barr virus; BCG, Bacillus Calmette-Guérin; HBV, hepatitis B virus; IgG, immunoglobulin G; MMR, measles vaccine; NA, not available; OR, odds ratio; VCA, viral capsid antigen.

^*^ The odds ratio of season of birth was based on spring vs. autumn. I^2^ presents the heterogeneity across included studies for each meta-analysis.

The search strategy in PubMed was listed as following: **study design**-"Meta-Analysis" [Publication Type] or meta-analysis[tiab] or meta analysis[tiab] or "Systematic Review" [Publication Type] or systematic review[tiab]; **outcome**-"Multiple Sclerosis"[Mesh] or Disseminated Sclerosis[ti] or Multiple Sclerosis[ti]; **overall** **exposures**-"Risk Factors"[Mesh] or Risk Factor[tiab]; **individual factor**-sun exposure[tiab] or "Sunlight"[Mesh] or sunshine[tiab] or sunlinght[tiab]; Latitude[tiab]; Season of birth[tiab]; Solar and geomagnetic activity[tiab]; Pesticides[Mesh] or Pesticide-related products [tiab]; "Air Pollution"[Mesh] or air pollution[tiab]; Exposure to organic solvents[tiab]; Farm animals and pets[tiab]; Epstein-Barr Virus Infections[Mesh] or EBV infection[tiab]; ERV reactivation[tiab]; Cytomegalovirus Infections[Mesh] or Cytomegalovirus infection[tiab]; Vaccines[Mesh] or vaccines[tiab] or "Vaccination"[Mesh] or vaccination[tiab]; Toxoplasmosis[Mesh] or Toxoplasma gondii infection[tiab]; Anti-Bacterial Agents[Mesh] or Antibiotic use[tiab] or antibiotic[tiab]. We used two-step search method: step 1(study design AND outcome AND exposures) and step 2 (study design AND outcome AND individual factor). There was no meta-analysis on the associations of sun exposure, pesticide-related products, air pollution, farm animals and pets and antibiotic use with risk of multiple sclerosis. For certain risk factors with more than one meta-analysis, we included the latest paper with the most included studies.

**References**

1. Hemani G, Zheng J, Elsworth B, Wade KH, Haberland V, Baird D, Laurin C, Burgess S, Bowden J, Langdon R *et al*: The MR-Base platform supports systematic causal inference across the human phenome. *Elife* 2018, 7.

2. Burgess S, Davies NM, Thompson SG: Bias due to participant overlap in two-sample Mendelian randomization. *Genet Epidemiol* 2016, 40(7):597-608.

3. Brion MJ, Shakhbazov K,Visscher PM: Calculating statistical power in Mendelian randomization studies. *Int J Epidemiol* 2013,42:1497-1501

4. Beecham AH, Patsopoulos NA, Xifara DK, Davis MF, Kemppinen A, Cotsapas C, Shah TS, Spencer C, Booth D, Goris A *et al*: Analysis of immune-related loci identifies 48 new susceptibility variants for multiple sclerosis. *Nat Genet* 2013, 45(11):1353-1360.

5. Simpson S, Jr., Wang W, Otahal P, Blizzard L, van der Mei IAF, Taylor BV: Latitude continues to be significantly associated with the prevalence of multiple sclerosis: an updated meta-analysis. *J Neurol Neurosurg Psychiatry* 2019, 90(11):1193-1200.

6. Abdollahi F, Sajedi SA: Correlation of multiple sclerosis (MS) incidence trends with solar and geomagnetic indices: Time to revise the method of reporting MS epidemiological data. *Iran J Neurol* 2014, 13(2):64-69.

7. Pantavou KG, Bagos PG: Season of birth and multiple sclerosis: a systematic review and multivariate meta-analysis. *J Neurol* 2019.

8. Barragan-Martinez C, Speck-Hernandez CA, Montoya-Ortiz G, Mantilla RD, Anaya JM, Rojas-Villarraga A: Organic solvents as risk factor for autoimmune diseases: a systematic review and meta-analysis. *PLoS One* 2012, 7(12):e51506.

9. Almohmeed YH, Avenell A, Aucott L, Vickers MA: Systematic review and meta-analysis of the sero-epidemiological association between Epstein Barr virus and multiple sclerosis. *PLoS One* 2013, 8(4):e61110.

10. Sundqvist E, Bergstrom T, Daialhosein H, Nystrom M, Sundstrom P, Hillert J, Alfredsson L, Kockum I, Olsson T: Cytomegalovirus seropositivity is negatively associated with multiple sclerosis. *Mult Scler* 2014, 20(2):165-173.

11. Sestili C, Grazina I, La Torre G: HBV vaccine and risk of developing multiple sclerosis: a systematic review and meta-analysis. *Hum Vaccin Immunother* 2018 [Online ahead of print].

12. Farez MF, Correale J: Immunizations and risk of multiple sclerosis: systematic review and meta-analysis. *J Neurol* 2011, 258(7):1197-1206.

13. Saberi R, Sharif M, Sarvi S, Aghayan SA, Hosseini SA, Anvari D, Nayeri Chegeni T, Hosseininejad Z, Daryani A: Is Toxoplasma gondii playing a positive role in multiple sclerosis risk? A systematic review and meta-analysis. *J Neuroimmunol* 2018, 322:57-62.
